# Sodium bicarbonate and intubation in severe diabetic ketoacidosis: are we too quick to dismiss them?

**DOI:** 10.1186/s40842-024-00171-y

**Published:** 2024-04-15

**Authors:** Manudi Vidanapathirana

**Affiliations:** Professorial Medical Unit, National Hospital of Sri Lanka, Colombo, Sri Lanka

**Keywords:** Diabetic ketoacidosis, Sodium bicarbonate, Intubation, Refractory acidosis

## Abstract

Management of diabetic ketoacidosis (DKA) has internationally established guidelines. However, management of severe, refractory DKA with multiple contributors to acidosis, and management of DKA in patients with altered mentation, remain ambiguous. Use of sodium bicarbonate and intubation in DKA are unpopular treatment practices, but warrant consideration in these unique clinical scenarios. This paper describes a 61-year-old Sri Lankan female who presented with severe DKA, seizures and altered level of consciousness. In her case, the acidosis was secondary to DKA, hyperlactatemia, hyperchloraemic acidosis and acute kidney injury (AKI). Intravenous sodium bicarbonate was used in the management of acidosis. She was intubated due to altered level of consciousness with inadequate respiratory drive to compensate for metabolic acidosis. The outcome in her case was favorable. Intravenous sodium bicarbonate in DKA should be considered for patients with severe, refractory acidosis with hemodynamic instability, hyperkalemia and compounding acidosis due to normal anion gap acidosis or AKI. Intubation should be considered for patients with obtunded mentation unable to achieve respiratory compensation and obtunded mentation where reversal of DKA is unlikely to improve consciousness. Both strategies should be personalized with consideration of individual risk vs benefit.

## Introduction

There are clearly established international guidelines for management of diabetic ketoacidosis (DKA). In these guidelines, fluid resuscitation and insulin therapy appear at the forefront of management [1–3]. However, there are clinical scenarios when DKA is severe and refractory, and complicated with alternate pathologies contributing to acidosis, and altered level of consciousness. Management of these complex clinical scenarios are not clearly outlined in guidelines. In such instances, unpopular management options such as the use of sodium bicarbonate and mechanical ventilation may need to be considered.

Use of sodium bicarbonate is generally frowned upon in the management of DKA. United States (US) guidelines recommend its use if pH is < 6.9 [1]. However, multiple studies have found no overall benefit of the use of sodium bicarbonate in patients with DKA. Studies have in fact, identified multiple risks of its use [4]. Intubation in DKA is also associated with multiple risks including cardiac arrest during intubation. Existing guidelines are not clear on the indications for intubation in DKA and the dos and don’ts of intubation in DKA [5].

This case report will describe the case of a 61-year-old Sri Lankan female who presented with severe DKA, seizures and altered level of consciousness. She had refractory acidosis with combined normal and high anion gap acidosis. Contributors to acidosis in her case were DKA, hyperlactatemia, hyperchloraemic acidosis due to use of normal saline and acute kidney injury (AKI).

Two questions arose during the management of this patient. One was the place for early initiation of sodium bicarbonate in DKA, as she had refractory acidosis with haemodynamic instability and alternate contributors to acidosis apart from DKA. The second question, was the place for intubation in DKA.

The author hopes that the discussion that follows will answer questions regarding these ambiguous areas in DKA management, and will generate more studies leading to changes in management practices.

## Case presentation

A 61-year-old Sri Lankan female presented to the emergency department with several episodes of vomiting and drowsiness over 6 h. There was no fever, headache or an identifiable focus of infection. There was no trauma to the head and no history of toxin ingestion.

Her comorbidities included ketosis prone diabetes mellitus, bronchial asthma and hypertension. There was a recent history of missing insulin doses.

On examination, her Glasgow Coma Score (GCS) was 5/15 and pupils were bilaterally reactive. There was no recorded temperature and no neck stiffness. Pulse rate was 101 beats per minute with cold peripheries and a blood pressure of 92/58 mmHg. Lungs were clear with a saturation of 96% on room air. She was hyperpnoeic with a respiratory rate was 32 cycles per minute. The abdomen was soft and a limited neurological examination revealed no focal neurological deficits.

Capillary blood sugar on admission was high index. Initial venous blood gas (VBG) showed a severe high anion gap metabolic acidosis, with serum ketone bodies of 6 mmol/L. Thus, a diagnosis of severe DKA was established and management was started according to standard guidelines with fluid resuscitation and insulin infusion. The blood gas trends during the management are shown in Table 1.

**Table 1:** Venous blood gas trends during initial resuscitation

	**On admission**	**After 1 h**	**After 2 h**	**After 4 h**
pH	6.654	6.674	6.93	7.11
PaCO2 (mmHg)	15	30.4	7.0	6.9
HCO3- (mEq/L)	1.5	4.5	1.5	2.3
Glucose (mg/dL)	Overload	Overload	512	470
Lactate (mmol/L)	4.8	3.6	3.2	2.4
Na + (mmol/L)	135 (Corrected Na + 142	149	141	139
K + (mmol/L)	7.41	3.49	3.16	3.29
Cl (mmol/L)	96	111	108	115
Anion gap (mEq/L)	30	37	30	20.8
Delta gap	1.18	1.1	0.8	0.4

*PaCO2* Partial pressure of carbon dioxide

In the first hour, important treatments were fluid resuscitation with 30 ml/hour of normal saline, insulin infusion at 6 units per hour and standard hyperkalaemia management.

In the second hour, she was started on inotropic support with noradrenaline due to persistent haemodynamic instability and the insulin infusion was increased to 8 units/hour due to inadequate decrease in glucose. Additionally, in the second hour, she developed 2 episodes of generalized tonic–clonic seizures which were managed with benzodiazepines and intravenous leveteracetam.

By the third hour, she was haemodynamically stable on inotropes, but glucose level was still high. The insulin infusion was increased to 12 units per hour. There was no improvement in in the GCS and respiratory rate was persistently high (30–40 cycles per minute). The VBG at the third hour showed combined normal and high anion gap metabolic acidosis; this was attributed to be due to fluid resuscitation with 0.9% NaCl. Considering the contribution by normal anion gap acidosis (NAGMA), intravenous 8.4% sodium bicarbonate of 100ml was given over 1 h.

After 4 h of management at the emergency department, the patient was escalated for care at the Intensive Care Unit (ICU). She was intubated due to persistently low GCS and ventilated with synchronized intermittent mandatory ventilation support. Additionally, she was treated with isotonic sodium bicarbonate infusion of 50 ml per hour due to the ongoing NAGMA and newly identified AKI. Summary of her investigations are shown in Table 2.

**Table 2:** Summary of investigations

	**Day 1**	**Day 2**	**Day 3**	**Day 4**	**Day 6**
WBC (× 1000/UL)	16.85	12.56	7.71	6.79	4.8
Hb (g/dL)	11.2	12.7	10.6	10.4	10.1
PLT (× 1000/UL)	253	221	100	66	154
S.Cr (mg/dL)	2.31	2.09	1.32	0.85	0.78
Na + (mmol/L)	140	157	151	146	145
K + (mmol/L)	4.6	4.7	3.2	4.0	3.5
CRP (mg/dL)	7.9	55	133	98	28
AST (U/L)	35		666	598	145
ALT (U/L)	18		185	279	124
Bilirubin (mg/dl)	0.1		0.6	1.0	1.2
Calcium (mg/dL)	10.7				
INR		0.91	1.18		
UFR	Pus cells-30–45 Red cells-5–6				
Urine culture	Mixed bacterial growth				
Blood culture	No growth				
NCCT brain	Normal				

*WBC* White cell count, *Hb* Haemoglobin, *PLT* platelet count, *S.Cr* serum creatinine, *CRP* C Reactive Protein, *INR* International Normalised Ratio, *NCCT* Non Contrast Computed Tomography, *UFR* Urine Full Report

The patient recovered from DKA 24 h after ICU care, and was extubated 48 h later.

Precipitants for DKA in this admission were poor compliance with insulin and urosepsis. The sepsis was managed with intravenous piperacillin tazobactam.

## Discussion

The discussion will explore the indications for two unpopular management options in DKA: the place for early initiation of sodium bicarbonate and the place for intubation.

### Place for sodium bicarbonate in management of DKA

Use of intravenous bicarbonate in DKA and alternate illnesses with metabolic acidosis is controversial. Problems associated with bicarbonate use are reduction of oxygen delivery to tissues by causing a left shift of the oxygen dissociation curve, paradoxical increase in intracellular acidosis and cerebral spinal fluid acidosis, increased risk of hypokalemia, cerebral oedema, delay in the fall in blood lactate: pyruvate ratio and delay in the fall of ketones [2, 6]. On the other hand, the problems with allowing acidosis to persist are negative cardiovascular and pulmonary effects due to myocardial depression, decreased systemic vascular tone, catecholamine resistance and pulmonary vasoconstriction [4]. There is also a resultant impairment of the immune response and leucocyte function, increasing the risk of infection [4].

While there is conflicting evidence for the use of sodium bicarbonate in metabolic acidosis, there seem to be multiple studies that show benefit of its use in instances of AKI.

A multicentre randomized controlled trial conducted among 389 ICU patients with severe metabolic acidosis (pH <  = 7.2) revealed no reduction in mortality or organ failure with sodium bicarbonate, but in a certain stratum of patients with AKI, there was reduction of day 28 mortality and need for renal replacement therapy [6]. Furthermore, there were no life-threatening complications related to the use of sodium bicarbonate in these patients, although metabolic derangements (hypernatremia, hypokalaemia and hypocalcaemia) were more frequently noted in the bicarbonate group [6].

A study of 1718 septic patients carried out by Zhang et al., further supports the use of sodium bicarbonate in septic patients with AKI (stages 2 and 3) and severe acidosis with a pH less than 7.2 [7]. Improved survival was seen in these specific groups of patients with sodium bicarbonate, although this survival benefit was not carried over to patients with pH >  = 7.2 and stage 1 AKI [7].

Similarly, a meta-analysis carried out in 2019, also shows weak evidence for a possible mortality benefit and improved renal outcomes with sodium bicarbonate in critically ill patients with AKI [8].

With regard to the use of bicarbonate in DKA, a handful of studies are available, but there are no randomized control trials with adequately powered sample sizes [9–19]. These studies concluded a lack of benefit with the use of sodium bicarbonate in DKA with severe acidosis, but only with regard to mortality and the time for resolution of acidaemia [9–19]. It is unclear whether bicarbonate may have improved haemodynamic stability and cardiac-related morbidity in these patients. Furthermore, these studies had very small sample sizes, used variable dosing regimens of bicarbonate, and lacked consistent data with regard to alternate contributors to acidosis. A summary of the studies on the use of bicarbonate in DKA is given in Table 3.

**Table 3:** Summary of studies on the use of sodium bicarbonate in DKA

Study	Year	Type of study	Study Population	Outcome	Confounders
Treatment of severe diabetic ketoacidosis. A comparative study of two methods [9]	1979	Interventional study	24 patients with severe diabetic ketoacidosis (pH < 7.10	Fall of plasma glucose concentration, rise in arterial pH and decrease in 3-hydroxybutyrate were similar in both groups	• Difference in the insulin regime used in the two groups • Small sample size
Sodium bicarbonate therapy in severe diabetic ketoacidosis [10]	1983	Retrospective analysis	95 patients with severe DKA	Rates of recovery of plasma glucose, bicarbonate levels, pH, and level of consciousness were similar in both groups	• Variable pHs in patients • No consideration of alternate contributors to acidosis
Metabolic effects of bicarbonate in the treatment of diabetic ketoacidosis [11]	1984	Randomized study (Bicarbonate vs normal saline)	32 patients	Use of bicarbonate delayed the fall in blood lactate, lactate: pyruvate ratio and total ketone bodies	• Small sample size • Variable pHs in the patients • No assessment of the alternate contributors to acidosis
Bicarbonate therapy in severe diabetic ketoacidosis [12]	1986	Randomized prospective protocol	21	No significant differences in the rate of decline of glucose or ketone levels or in the rate of increase in pH or bicarbonate levels in the blood or cerebrospinal fluid in either group	• Variable pHs in the patients • No assessment of the alternate contributors to acidosis • Variable dosing of bicarbonate
Bicarbonate therapy in severe diabetic ketoacidosis. A double blind, randomized, placebo controlled trial [13]	1991	Double-blind, randomized, placebo controlled trial (Bicarbonate vs normal saline)	9	In patients with severe DKA (pH < 7.15), no clinical or metabolic differences in the 2 groups were seen	• Small sample size • No assessment of the alternate contributors to acidosis
Counterproductive effects of sodium bicarbonate in diabetic ketoacidosis [14]	1996	Randomized study (Bicarbonate vs placebo)	7 patients	Alkali administration augmented ketone production	• Small sample size • No assessment of alternate contributors to acidosis • Ambiguous dosing of bicarbonate
Does bicarbonate therapy improve the management of severe diabetic ketoacidosis? [15]	1999	Randomized study (Bicarbonate vs placebo)	39	In patients with severe DKA, with pH between 6.90 -7.1, there was no difference in normalization of clinical or biochemical parameters	• Small sample size • No assessment of the alternate contributors to acidosis
Diabetic ketoacidosis and bicarbonate therapy [16]	2000	Retrospective study	39 patients with severe DKA	Bicarbonate in patients with severe DKA does not produce a more rapid normalisation of biochemical or clinical parameters	• Retrospective nature of the study • No assessment of the alternate contributors to acidosis • Variable dosing of bicarbonate
Intravenous sodium bicarbonate therapy in severely acidotic diabetic ketoacidosis [17]	2007–2011	Retrospective cohort study	86	Intravenous bicarbonate therapy did not decrease time to resolution of acidosis or time to hospital discharge for patients with DKA with initial pH < 7	• No data on ketone or lactate levels • Use and dosing of bicarbonate was dictated by physician preference
Bicarbonate in diabetic ketoacidosis – a systematic review [18]	2011	Systematic Review of 44 articles, including 3 randomized controlled trials		No benefit of sodium bicarbonate in the emergency treatment of DKA	No inclusion of patients with a pH of < 6.85
Sodium bicarbonate is safe but not useful in the management of severe diabetic ketoacidosis [19]	2018–2022	Retrospective study	232 patients aged 1 month-18 years with pH < 6.9	• Length of stay in the PICU, insulin infusion duration, and acidosis recovery time were significantly higher in the bicarbonate group • Sodium bicarbonate had no significant effect on respiratory and heart rates, pH, PaCO2, anion gap, and bicarbonate level • Improvement of GCS was greater in the bicarbonate group	• No assessment of the alternate contributors to acidosis • Variable dosing of bicarbonate

Considering the harmful consequences of ongoing metabolic acidosis, some DKA guidelines such as the US guideline and the Sri Lankan guideline make a case in favour of the use of sodium bicarbonate in severe refractory acidosis [1, 3]. Furthermore, it appears that the use of bicarbonate in DKA arises in instances with alternate contributors to acidosis, such as in AKI and hyperchloraemic NAGMA [4]. None of the studies mentioned in Table 3 have adequately looked into the occurrence of these causes as contributors to acidosis and the use of bicarbonate in these instances. Therefore, there is still a dearth of adequate research with randomized controlled trials, using proper patient selection, in this area.

From the available evidence, there is a potential role for sodium bicarbonate in the recovery phase of DKA for these two indications: in instances of hyperchloraemia due to fluid resuscitation that will offset the correction of acidosis resulting from DKA resolution and in the presence of AKI [4].

It is difficult to comment on whether sodium bicarbonate played a role in the improvement of this patient, who had compounding AKI and NAGMA, in addition to ketoacidosis. In either case, it certainly raises interesting questions with regard to the place for sodium bicarbonate in DKA.

The author feels that while fluid resuscitation and insulin therapy must remain at the forefront of DKA management, sodium bicarbonate may be considered (not necessarily always given), early rather than late, in patients that have severe metabolic acidosis with haemodynamic instability, AKI, NAGMA and hyperkalemia. Calculation of the delta ratio must therefore be routinely done to identify co-existent NAGMA.

The recommended administration of sodium bicarbonate is 50 mmol in 200 ml of sterile water to be administered over 1 h [6]. This can be doubled and infused over 2 h until pH is > 7 [6]. 10 mmol of KCL can also be considered to be added to each 200 ml due to the expected hypokalaemia with sodium bicarbonate [6].

### Place for intubation in DKA

Patients with DKA are at risk of respiratory failure due to pneumonia, acute respiratory distress syndrome (ARDS) and pulmonary edema [20]. Two types of pulmonary edema have been recognized in DKA: one due to raised pulmonary venous pressure and one due to increased capillary permeability [20].

During DKA, patients develop hyperventilation as a compensatory mechanism, and once they reach the point of Kussmaul’s breathing, they are at risk of respiratory muscle fatigue and ARDS, due to hyperpnoea [16]. Furthermore, a certain percentage of patients who are comatose either due to the severity of DKA or due to an alternate insult, may be unable to generate respiratory alkalosis sufficient enough to compensate for the existing metabolic acidosis [20]. These patients are candidates to be considered for intubation.

However, multiple problems exist with intubation of DKA patients. These are presence of acidosis and hypotension in DKA, worsening of acidosis during sedation and/or paralysis due to rise in carbon dioxide, high risk of aspiration due to gastroparesis and vomiting, possible worsening of hyperkalaemia when using succinylcholine for rapid sequence intubation, difficulty matching the degree of respiratory compensation with ventilator settings and risk of lung injury with high pressure ventilator settings [20, 22].

Steps to overcome the above are [20–22]:

1. Best possible resuscitation with fluids, insulin and if indicated sodium bicarbonate prior to intubation
2. Placement of a nasogastric tube and aspiration prior to intubation
3. Rapid sequence intubation with cricoid pressure
4. Use of drugs with minimal effects on cardiovascular stability
5. Tidal volume of 8 ml/kg based on ideal body weight and respiratory rate similar to the patient’s compensating respiratory rate

The author suggests consideration of intubation for patients with obtunded mentation unable to achieve respiratory compensation and alternate indications for intubation (for example, status epilepticus or alternate pathologies that hinder recovery of GCS despite resolution of DKA). However, it is important to bear in mind the risks of intubation and follow the above steps in order to mitigate said risks.

## Conclusion

Use of sodium bicarbonate and intubation in DKA have remained poorly explored treatment areas in literature and guidelines. The use of sodium bicarbonate in DKA should be considered for patients with severe refractory acidosis with hemodynamic instability, hyperkalemia and alternate contributors for acidosis through NAGMA or AKI. The author recommends routine calculation of the delta ratio to help guide management in this regard. Consideration of intubation should be reserved for patients unable to achieve respiratory compensation due to obtunded mentation and patients with altered level of consciousness due to alternate pathologies where DKA reversal is unlikely to improve GCS. Both treatment strategies should be considered on an individual basis with a careful reflection of risk vs benefit.

## Data Availability

All data generated or analyzed during this study are included in this published article [and its supplementary information files].

## Abbreviations

AKI: Acute Kidney Injury
CRP: C Reactive Protein
DKA: Diabetic ketoacidosis
GCS: Glasgow Coma Score
Hb: Haemoglobin
ICU: Intensive Care Unit
INR: International Normalised Ratio
NAGMA: Normal Anion Gap Metabolic Acidosis
NCCT: Non Contrast Computed Tomography
PaCO2: Partial pressure of carbon dioxide
PLT: Platelet count
S.Cr: Serum creatinine
UFR: Urine Full Report
US: United States
VBG: Venous Blood Gas
WBC: White Blood cell Count
